# Material Characterization and Electrochemical Properties of Titanium Alloy 5553 Prepared by Selective Laser Melting as Processed and after Abrading and Polishing

**DOI:** 10.1021/acsomega.3c08935

**Published:** 2024-07-25

**Authors:** Isuri N. Dammulla, Ryan Weston, Zia Uddin Mahmud, Sujoy Saha, Sarah McFall-Boegeman, Luke Rice, Jonathan H. Dwyer, Taylor Kmetz, Carl J. Boehlert, Greg M. Swain

**Affiliations:** † Department of Chemistry, 578 South Shaw Lane, Chemistry Building, Michigan State University, East Lansing, Michigan 48824-1322, United States; ‡ Department of Chemical Engineering and Material Science, 428 South Shaw Lane, Engineering Building, Michigan State University, East Lansing, Michigan 48824-4437, United States; § Department of Energy’s Kansas City National Security Campus Managed By Honeywell Federal Manufacturing and Technologies, LLC, 14520 Botts Road, Kansas City, Missouri 64147, United States

## Abstract

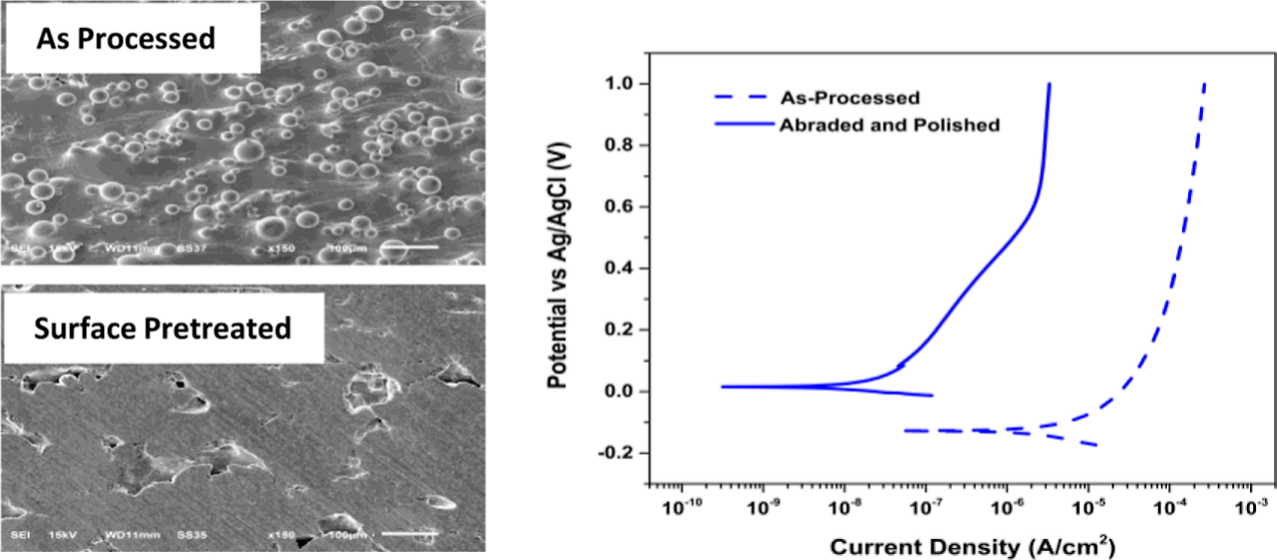

The microstructure, Vickers microhardness, and electrochemical properties of an additive manufactured titanium alloy, Ti-5553 (Ti–5Al–5Mo–5V–3Cr wt %), are reported on. The alloy specimens were fabricated by selective laser melt processing. The surface morphology and electrochemical properties of the as-processed and surface-pretreated (abraded and polished) Ti-5553 specimens were investigated. The as-processed specimens had a nominal density of 4.62 ± 0.04 g/cm^3^. Based on comparison with the reported density for the die-cast alloy, the specimens were 99–100% dense with ca. 1% porosity. Optical microscopy and scanning electron microscopy revealed some micropores, balling features, and fusion pore defects across the surface of the alloy (*XZ* plane—orthogonal to the build direction). The nominal Vickers microhardness was 292 ± 2 HV. Detailed electrochemical characterization of the as-processed and surface-pretreated alloys revealed reproducible open-circuit potentials (OCPs), linear polarization resistances (*R*_p_), and potentiodynamic polarization curves for both specimen types in naturally aerated 3.5 wt % NaCl at room temperature. For the surface-pretreated alloys, the OCP was 225 mV more noble, the anodic current in the potentiodynamic polarization curves was 72× lower, the cathodic current was 8× lower, and *R*_p_ was larger by 426× than the values for the as-processed specimens. The collective electrochemical data revealed that the surface-pretreated alloys exhibit greater corrosion resistance than the as-processed alloys due to a reduction of the real area and the formation of a more passivating oxide layer.

## 1 Introduction

Titanium alloys (Ti) are widely used in the aerospace (e.g., landing gear) and biomedical (e.g., implants) fields owing to their excellent mechanical properties such as high strength, high fracture resistance, good formability, high temperature properties, and corrosion resistance.^1−8^ The increased demand for these alloys and the high cost of conventional Ti components prepared using subtractive machining have driven interest in the bottom-up fabrication of intricate Ti parts needed for these applications using additive manufacturing (AM) techniques.^9−16^

AM is a process whereby parts and components are fabricated from the bottom up by using a layer-by-layer approach following a three-dimensional computer-aided design (3D CAD). This fabrication method contrasts with the conventional subtractive and formative manufacturing techniques, such as extrusion, forging, shape casting, machining, etc.^14−21^ The notable advantages of AM fabrication include the ability to design and prepare geometrically complex, lightweight structures with improved performance, reduced design-to-manufacture time, minimized waste production, and lower cost.^12−15,20,21^ Two broad classes of metal AM technologies are powder bed fusion (PBF) and directed energy deposition (DED). PBF methods enable manufacturing of geometrically complex products using a heat source, a laser, or electron beam to fuse powder particles layer by layer, forming a solid part. There are different types of PBF methods, including selective laser sintering, selective laser melting (SLM), and electron beam melting. DED AM technologies, on the other hand, inject the metal powders onto a substrate where a high energy density heat source, such as a laser, electron beam, or plasma electric arc, is focused, and these are more appropriate for manufacturing larger parts with a coarser finish.^9,17,20−22^ DED processes involve simultaneously adding material while heating.

SLM employs a high-power laser beam to raster-scan and melt the metal powder particles preplaced on a build platform. The melting of the regions of interest in a layer-by-layer manner fuses the molten metal into the desired structure upon cooling and solidification.^12−15,20−25^ This process is repeated until the final part geometry is achieved. The powder particle fusion depends on multiple process variables, including laser power, scanning speed, scanning pattern, and part geometry. These parameters influence the size of the melt pool around the scanning laser beam and the thermal gradient experienced by nearby particles.^15^ Ti–6Al–4V (wt %) is the most prepared SLM Ti alloy as reflected in the scientific literature, some of which is cited here.^26−43^ There has been much less work on preparing Ti-5553 alloys by this AM method. Therefore, much less is known about the structure–property relationships compared to Ti–6Al–4V.

Ti–5Al–5V–5Mo–3Cr (wt %) (Ti-5553) alloys are of interest for aerospace applications due to the material’s high strength, low weight, and good resistance to fatigue and crack propagation. This near-β phase alloy exhibits good castability and weldability.^44,45^ This alloy can be hardened through thermal treatment^45^ and can achieve strength up to 1300 MPa and elongation to failure values greater than 10%.^43,46^ Given these desirable mechanical properties, efforts are underway to prepare this alloy by using AM technologies. There is very little published on the structure–electrochemical property relationships of this SLM alloy, so this represents a significant knowledge gap.

Fundamental research is needed to better understand (i) how the fabrication parameters influence the material density, defects, microstructure, and electrochemical corrosion susceptibility and (ii) how different surface pretreatments and finishes can be optimally applied to mitigate corrosion. These structure–function relationships are not well-established for AM parts. Some work has been published describing the microstructure and mechanical properties of SLM-processed Ti-5553.^13,14,47−53^ However, there is little published research regarding the electrochemical characterization of SLM or die cast Ti-5553.^11,54−56^ To address this knowledge gap, we report herein on the material characterization and electrochemical properties of SLM-prepared Ti-5553 specimens as-processed (i.e., with their native surface roughness and oxide film) and after abrading and polishing to smooth the surface texture, thereby reducing the surface roughness and enabling the formation of a less defective and more compact oxide film.

## 2 Materials and Methods

### 2.1 Chemicals and Reagents

All of the chemicals used were of analytical grade quality or better. Sodium chloride (NaCl) was purchased from a commercial supplier (Sigma-Aldrich) and used as received. The Turco 6849 and Turco Liquid Smut-Go NC solutions were provided by Henkel Technologies, Inc. (Madison Heights, MI). Both were diluted with ultrapure water to 20% (v/v) before use. All aqueous solutions were prepared with ultrapure water (>17 Ω-cm) from a Barnstead E-Pure water purification system.

### 2.2 Fabrication of AM Ti-5553

An Additive Industries (AI) MetalFab1 laser PBF system was used to manufacture the specimens using Ti-5553 powder purchased from AP&C (Montreal, Canada). MetalFab1 uses four lasers, each of which is full field. The lasers were ytterbium (Yb)-doped fiber type with a maximum power of 500 W, a fundamental output wavelength of 1070 nm, and a spot size of 100–105 μm. The specimens were built via a continuous wave exposure strategy involving scan-path striping for each layer. Scan-path striping is a laser scanning strategy that divides the area to be consolidated into smaller sets of laser raster vectors. Each build layer is rotated 67° to avoid stacking scan corners and interior seams (this is a common build practice). The laser power during the build ranged from 120 to 160 W and the laser scan speed varied between 600 and 950 mm/s, depending on the cross-sectional geometry and layer. The powder layer thickness remained a constant 40 μm. All specimens were built with the long dimension perpendicular to the surface of the build plate to minimize the area needed to be cut for removal.

The parts were removed from the build plate by wire electrical discharge machining and cleaned by an in-house process, including media blasting, to remove excess powder. The media blast step removed semisintered particles from the specimen and provided a matte finish to the exterior. The specimens were sand-blasted at a pressure between 50 and 70 psi. Cleaning consisted of the following five steps: (i) high pressure spray with detergent followed by water rinsing, (ii) multifrequency (40–280 kHz) ultrasonication in detergent followed by water rinsing, (iii) ultrasonication (40 kHz) in deionized water and drying with a stream of N_2_ gas, (iv) vacuum oven drying (125 °C, 1 h, 10^–2^ Torr), and (v) packaging the cooled specimens into a nylon bag for shipment to Michigan State University. No thermal annealing after fabrication was applied to any of the parts. These specimens are referred to as processed.

Although the powder used for the alloy preparation is proprietary, it had the following general composition in wt % as indicated in Aerospace Material Specification AMS7026: Al (4.4–5.7), V (4.0–5.5), Mo (4.0–5.5), and Cr (2.5–3.5), with the balance as Ti. The particle size used in the builds had a diameter ranging from 20 to 63 μm. All specimens used in this work were 2.54 cm × 2.54 cm in dimension.

### 2.3 Digital Optical Microscopy

The surface texture of the as-processed Ti-5553 specimens, before and after smoothing by mechanical abrading and polishing, was investigated by using a VHX-6000 (Keyence Corp. USA) digital optical microscope. The as-processed specimens were abraded first with P1500 grit aluminum oxide grinding paper for 4 min on a polishing wheel and then ultrasonically cleaned in ultrapure water for 10 min. The specimens were then polished by hand for 5 min using 0.3 μm alumina powder (Buehler) slurried in ultrapure water on a felt pad. This was followed by ultrasonic cleaning in ultrapure water for 10 min. The specimens were then polished with 0.05 μm alumina powder (Buehler) slurried in ultrapure water on a separate felt pad by hand for 5 min. This was followed by ultrasonic cleaning in ultrapure water for 10–20 min. Optical microscopy was then used to image the specimens and to quantitatively assess the surface texture in terms of the root-mean square of the surface roughness (*S*_q_) and the maximum peak-to-valley height (*S*_z_). These values were calculated by analyzing five spots on three different specimens (area of a single spot = 1000 × 1000 μm^2^).

### 2.4 Scanning Electron Microscopy

Scanning electron microscopy (SEM) was performed by using a JSM-6610LV (JEOL USA Inc.) electron microscope. The as-processed specimens were characterized before and after abrading and polishing. The abrading and polishing steps were different from those used to smooth the specimens for optical microscopy. The alloys were initially wet-abraded by hand on P1500 grit aluminum oxide grinding paper for 4 min, followed by ultrasonic cleaning in ultrapure water for 20 min. They were then polished with a 0.25 μm alumina/H_2_O slurry on a polishing wheel for 30 min and rinsed with ultrapure water. They were next polished with a 0.04 μm colloidal silica/H_2_O slurry on a polishing wheel for 40 min, rinsed with ethanol, rubbed on a felt pad wetted with ethanol to remove the remaining polishing debris, and ultrasonically cleaned in ethanol for 10 min. Micrographs were collected at a working distance of 10 mm using an accelerating voltage of 25 kV. A smoother surface provides a better visualization of the grain boundaries, second-phase particles, microvoids, and other defects that might be present.

### 2.5 X-ray Diffraction Analysis

A 2.54 cm × 2.54 cm as-processed specimen was ultrasonically cleaned in ethanol for 15 min and then air-dried. A Rigaku SmartLab X-ray diffractometer was used to characterize the crystallographic structure of the specimen. A Cu (Kα) X-ray source (1.54 Å) was used at 40 kV and 44 mA over a 2θ scan range of 20–90 deg. The scan speed was 3.03°/min with a step width of 0.01°. The spot size was 0.4 × 12.0 mm.

### 2.6 Microhardness Measurements

The as-processed specimens were abraded, polished, and cleaned similar to the method of preparation used for the digital optical microscopy described above. The Vickers microhardness measurements were then performed on three smoothed specimens using the pyramidally shaped diamond indenter of a Clark CM-800AT microhardness tester. Measurements were made at 5 different spots (four corner regions and center) on each specimen by applying an indentation load of 200 gf for 15 s. The measurements and analysis were performed according to ASTM E384 (Standard Test for Microhardness of Materials).

### 2.7 Specimen Preparation Methods for Metallographic Analysis

The alloy specimens were first mechanically abraded on a polishing wheel with wet 12 and 8 μm aluminum oxide grinding papers, respectively, for 20 min each. This was followed by ultrasonic cleaning in ultrapure water (10 min) before sequentially polishing with 6, 3, 1, and 0.25 μm alumina/H_2_O slurries. Each polishing step was performed for 20 min on a polishing wheel. The specimens were rinsed with and ultrasonically cleaned in ultrapure water after each polishing step. The specimens were then polished with 0.04 μm colloidal silica for 40 min on a polishing wheel and rinsed with ethanol and ultrasonicated in ethanol for 10 min. Etching was then performed in Kroll’s solution by immersion of the sample for 30 s at room temperature. Kroll’s solution is designed specifically for Ti alloys. It has a composition of 6 wt % HNO_3_ and 1 wt % HF, with the balance as water. Care must be used with working with this etchant, as hydrofluoric acid is highly corrosive. Direct contact with the skin should be avoided using personal protective equipment. The acid dissociates into fluoride ions upon contact with the skin and these ions can cause damage to deep tissue layers and bone. After etching, the specimens were rinsed with ultrapure water and isopropanol, respectively, and dried with an N_2_ gas flow.

### 2.8 Electrochemical Measurements

The specimens were electrochemically characterized using open-circuit potential (OCP) measurements, linear polarization resistance (*R*_p_) measurements, anodic and cathodic potentiodynamic polarization curves, and full frequency electrochemical impedance spectroscopy (EIS) at the OCP. The electrolyte was naturally aerated 3.5 wt % NaCl. All electrochemical measurements were made at room temperature using a 1 cm^2^ flat cell (BioLogic Science Instruments, France) design in combination with a computer-controlled electrochemical workstation (Gamry Instruments, Inc., Reference 600, Warminster, PA). A specimen was mounted in the electrochemical cell against a Viton O-ring that defined the exposed geometric area, 1 cm^2^. There were no signs of significant crevice corrosion under the O-ring. All currents reported herein are normalized to the geometric area. The counter electrode was a Pt flag, and the reference was a homemade silver chloride electrode (Ag/AgCl, 4 M KCl, *E*° = 0.197 V vs NHE) that was isolated from the main solution in a Luggin capillary with a cracked glass tip.

The OCP was measured for at least 1 h after initial alloy contact with the electrolyte solution. Linear polarization resistance measurements were performed using linear sweep voltammetry over a Δ*E* of ±20 mV relative to the OCP. The scan rate was 1 mV/s. The reciprocal slope of the *i*–*E* curve is the polarization resistance, *R*_p_.^57^

Potentiodynamic polarization curves were recorded from ±0.050 V vs OCP to either a positive limit of 1.0 V vs Ag/AgCl for the anodic curves or to a negative limit of −1.0 V vs Ag/AgCl for the cathodic curves. The scan rate was 1 mV/s. EIS measurements were made at the OCP using a 10 mV sine wave with seven points per decade of frequency recorded for analysis. A range from 10^5^ to 10^–2^ Hz was employed to determine the frequency dependence of the real (ohmic) and imaginary (capacitive) components of the total impedance. The experimental data were analyzed by fitting to an appropriate equivalent circuit using ZView software (version 3.5a). This was performed to determine the numerical magnitudes of the circuit components (i.e., electrochemical parameters). All electrochemical measurements were repeated with at least three specimens of each type (as-processed and surface-pretreated) to assess response reproducibility.

Electrochemical analysis was performed using as-processed and surface-pretreated specimens. The as-processed specimens were only degreased and deoxidized prior to use in electrochemical measurements. The surface-pretreated specimens were smoothed by abrading and polishing, as described above for digital optical microscopy, as well as degreased and deoxidized.

The degreasing was by immersion in an alkaline cleaner (Bonderite C-AK 6849 Aero, Henkel Technologies) for 10 min at 55 °C followed by a 2 min flowing city tap water rinse. The deoxidation was by immersion in a commercial solution (Bonderite C-IC Smut-Go NC Aero, Henkel Technologies) for 2 min at room temperature. The specimens were rinsed again in flowing city tap water for 2 min and dried with a low pressure N_2_ gas flow. All specimens were used immediately afterward for the electrochemical measurements.

## 3 Results

### 3.1 Density Measurements

The density of as-processed Ti-5553 specimens was determined from (i) weight and volume and (ii) water volume displacement measurements. The nominal density for five different specimens determined by both methods was 4.62 ± 0.04 g/cm^3^. This value is slightly lower than the density reported for the fully dense die-cast alloy of 4.65 g/cm^3^.^48,58^ Therefore, the SLM alloys are near fully dense, with an estimated porosity of ∼1%.

### 3.2 Specimen Texture and Morphology

**Figure 1:**
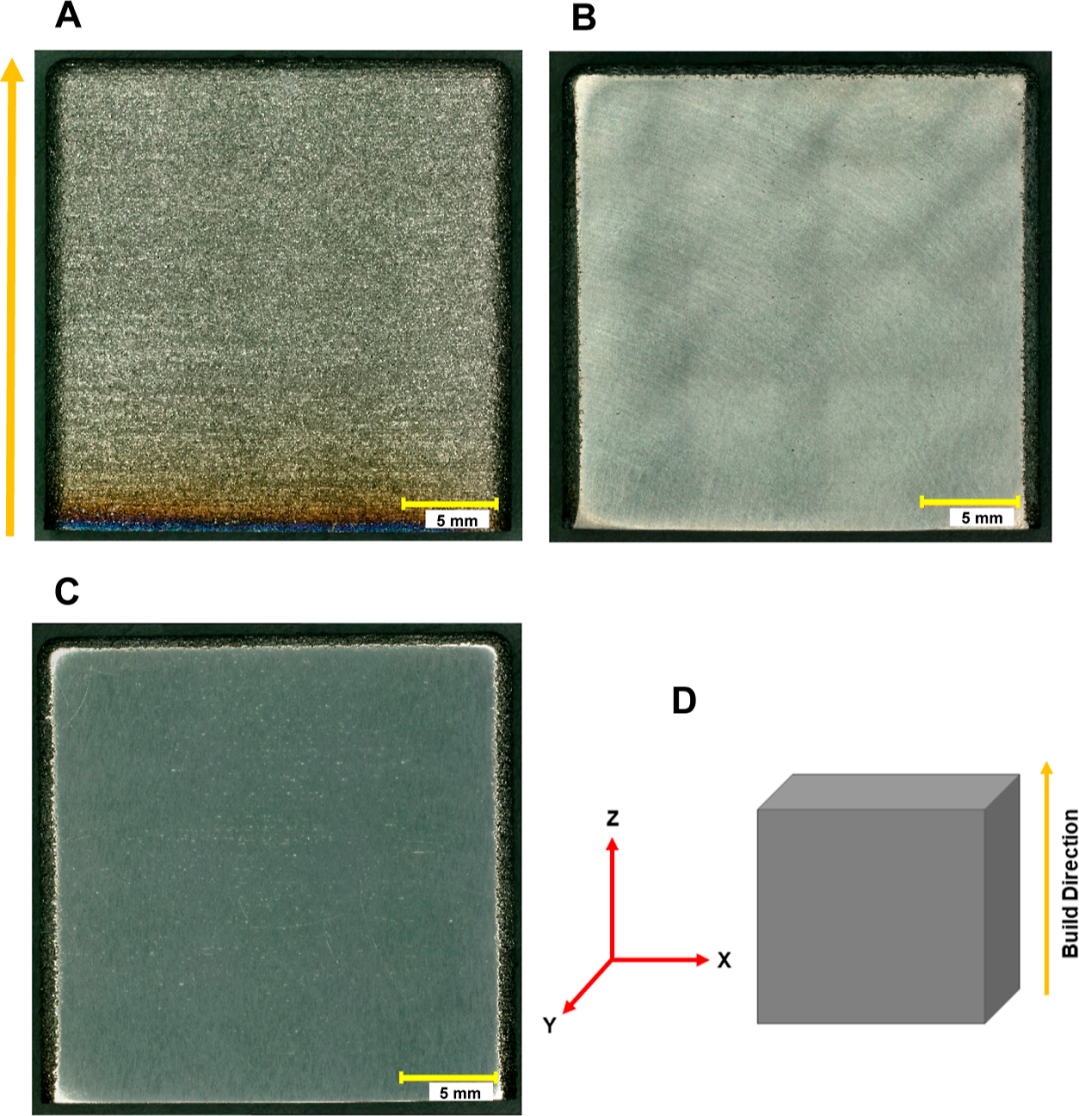
Digital optical micrographs of a typical Ti-5553 specimen (A) as-processed, (B) after mechanical abrading on wet P1500 grit aluminum oxide grinding paper, and (C) after mechanical abrading and polishing with decreasing grades of alumina powder. The full 2.54 × 2.54 cm^2^ specimen is shown. The yellow arrow on the left shows the build direction. The micrographs are of the *XZ* plane orthogonal to the build plane. The scale bar in the micrographs is 5 cm. (D) Schematic diagram indicating the build direction and *X*, *Y*, and *Z* axes.

Figure 1 presents the optical micrographs of a typical as-processed specimen surface before and after abrading on wet P1500 grit aluminum oxide grinding paper and polishing with decreasing grades of alumina powder (see Section 2.3). The yellow arrow in Figure 1A indicates the build direction. Before any abrading and polishing (Figure 1A), the alloy consists of a rough surface texture characteristic of the SLM fabrication process. The as-processed specimen surface roughness was reduced significantly by the abrading and polishing steps. The abrading introduced striations that are visible in the micrograph running from the bottom to the left of the specimen (Figure 1B). A smoother and striation-free surface resulted after abrading and polishing (Figure 1C). Figure 1D illustrates the build direction and the *X*, *Y*, and *Z* axes of the specimens.

**Figure 2:**
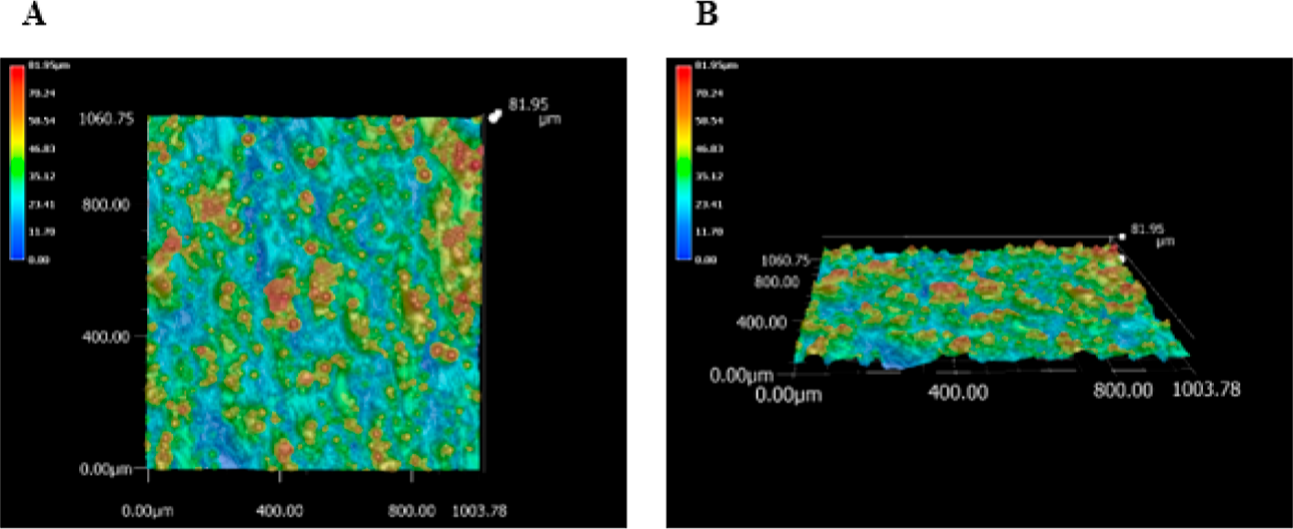
Height color maps of the surface topography of an as-processed Ti-5553 alloy specimen: (A) top view and (B) 3D contour plot. The maps were generated from 3D images collected at 1000×. The *XZ* plane is shown.

Figure 2A,B shows the height color plots of the surface topography of the same region of an as-processed specimen (Figure 1A). The plots reveal a rough surface texture with the red regions being raised features and the blue regions being depressions. The maximum in the *z*-axis color scale is 84 μm. Table 1 presents a summary of the surface texture analysis performed on the 3D raw images. The data are the arithmetic mean height (*S*_a_), maximum peak-to-valley height (*S*_z_), and surface roughness (*S*_q_). The nominal *S*_q_ value is 16.7 ± 5.3 μm.

**Table 1:** Surface Texture Analysis of the 3D Contour Data of As-Processed Ti-5553 Specimens^a^

surface texture parameter	
*S*_a_, arithmetical mean height	13.3 ± 4.3 μm
*S*_z_ maximum peak-to-valley height	111 ± 29 μm
*S*_q_, root-mean-square height (surface roughness)	16.7 ± 5.3 μm

a Data are reported as mean ± std. dev. for five spots on three different specimens. The 3D images were collected at 1000× and the surface texture data are for regions 1000 × 1000 μm^2^. Values were determined by using the Keyence microscope software.

**Figure 3:**
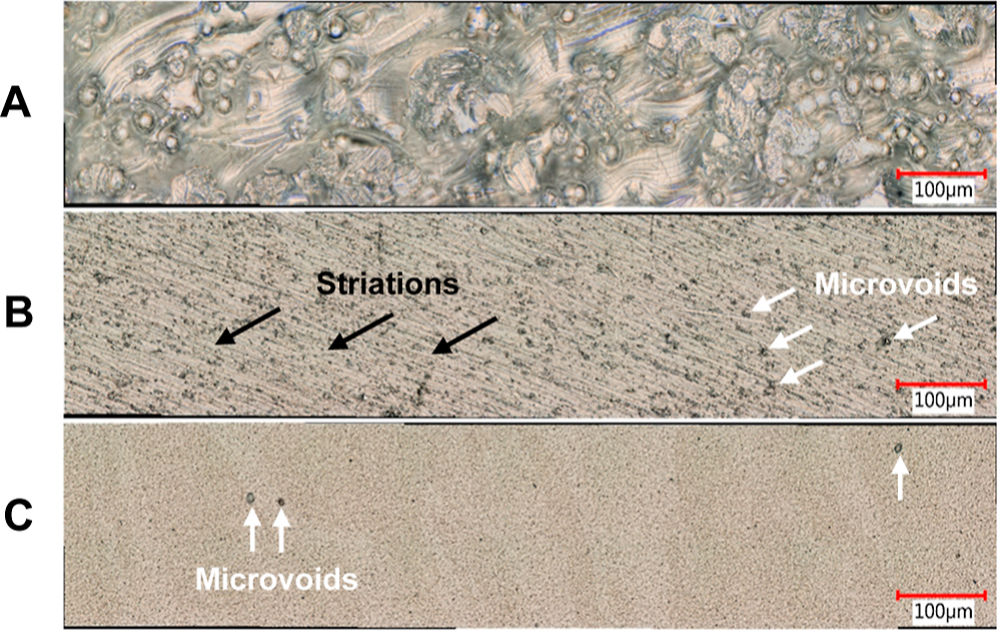
Optical micrographs of a Ti-5553 specimen: (A) as-processed, the (B) same specimen in A after mechanical abrading on wet P1500 grit aluminum oxide grinding paper using a polishing wheel, and the (C) same specimen in B after abrading and polishing by hand with decreasing grades of alumina powder. All micrographs were obtained at 2000× magnification. The *XZ* plane is shown. The scale bar in all the micrographs is 100 μm.

The 3D optical micrographs are presented in Figure 3 for a smaller area (1000 × 200 μm^2^) of the same alloy specimen presented in Figure 1. The micrographs reveal differences in the surface texture of the as-processed (Figure 3A), abraded (Figure 3B), and abraded and polished (Figure 3C) specimens (see Section 2.3). Table 2 summarizes the surface texture analysis data for a Ti-5553 specimen as processed and after abrading and polishing.

**Table 2:** Surface Texture Analysis of the 3D Image Data of the Ti-5553 Alloy Specimen in Figure 3 as Processed, after Abrading on a Wheel, and after Abrading on a Wheel and Polishing by Hand^a^

	as processed (μm)	after abrading on a polishing wheel (μm)	after abrading on a polishing wheel and polishing by hand (μm)
*S*_q_, root-mean-square height (surface roughness)	29 ± 7	1.8 ± 0.1	1.5 ± 0.1
*S*_z_ maximum peak-to-valley height	127 ± 25	6.4 ± 0.4	5.6 ± 0.2

a The table shows compiled data for *S*_q_ and *S*_z_ over five spots on a single specimen. Area of analysis = 1000 × 200 μm^2^. Values were determined using the Keyence microscope software.

The rough as-processed specimen exhibited some balling features (Figure 3A). This is a common defect in SLM materials that arises from competition between melt spreading and solidification during laser heating. In other words, melted droplets solidify before spreading completely to create a flat layer.^12−16,59^ Clearly, the surface was smoothed by abrading with P1500 grit alumina grinding paper on a mechanical polishing wheel, as both the surface roughness and maximum peak-to-valley height were reduced by an order of magnitude. The striations produced by the large grit mechanical abrading are apparent (Figure 3B). The striation widths of ca. 13 μm are consistent with the grit size of the P1500 grinding paper. The abrading revealed some microvoids and pores in the material that are not visible on the rough, as-processed specimen. Micropores are another type of defect commonly found in SLM alloys. Such defects result during the layer-by-layer build due to the entrapment of residual gas during the fabrication process.^12−15,19,20,59^ The trapped gas inhibits particle fusion and densification and leads to voids during the solidification process. The presence of the micropores is consistent with the ∼1% estimated porosity. Further polishing of the hard alloy by hand with 0.3 and 0.05 μm alumina grit did not lead to additional smoothing (Figure 3C). The alloy’s hardness is such that a few minutes of hand polishing with alumina powder do not alter the surface texture much.^60^

**Figure 4:**
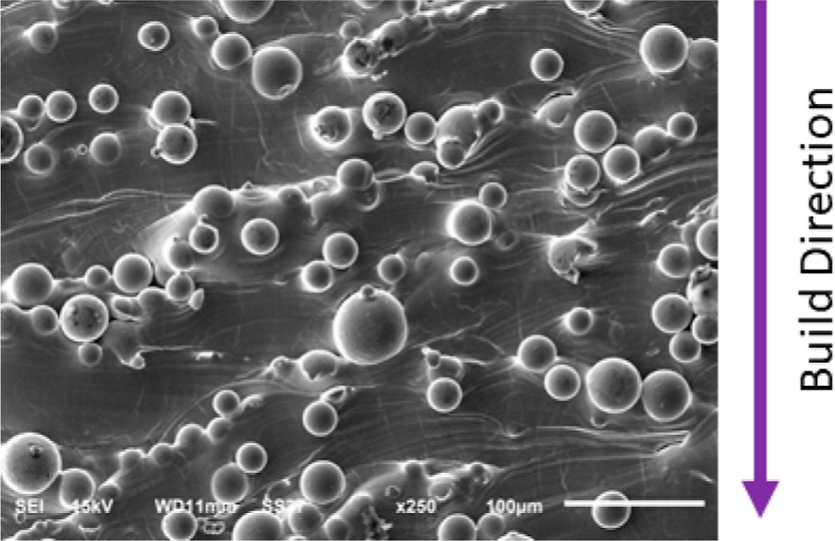
SEM micrograph (secondary electron image) of a typical as-processed Ti-5553 specimen. The scale bar is 100 μm. The *XZ* plane is shown.

Figure 4 presents a typical SEM micrograph of the surface of an as-processed Ti-5553 specimen. A rough surface texture is seen with partially and fully melted powder particles.

The ball features reflect powder particles that have been partially melted during the laser scanning process. These powder particles, although not fully melted, adhere to the surface. No micropores or cracks are evident on the rough surface.^13−16^

**Figure 5:**
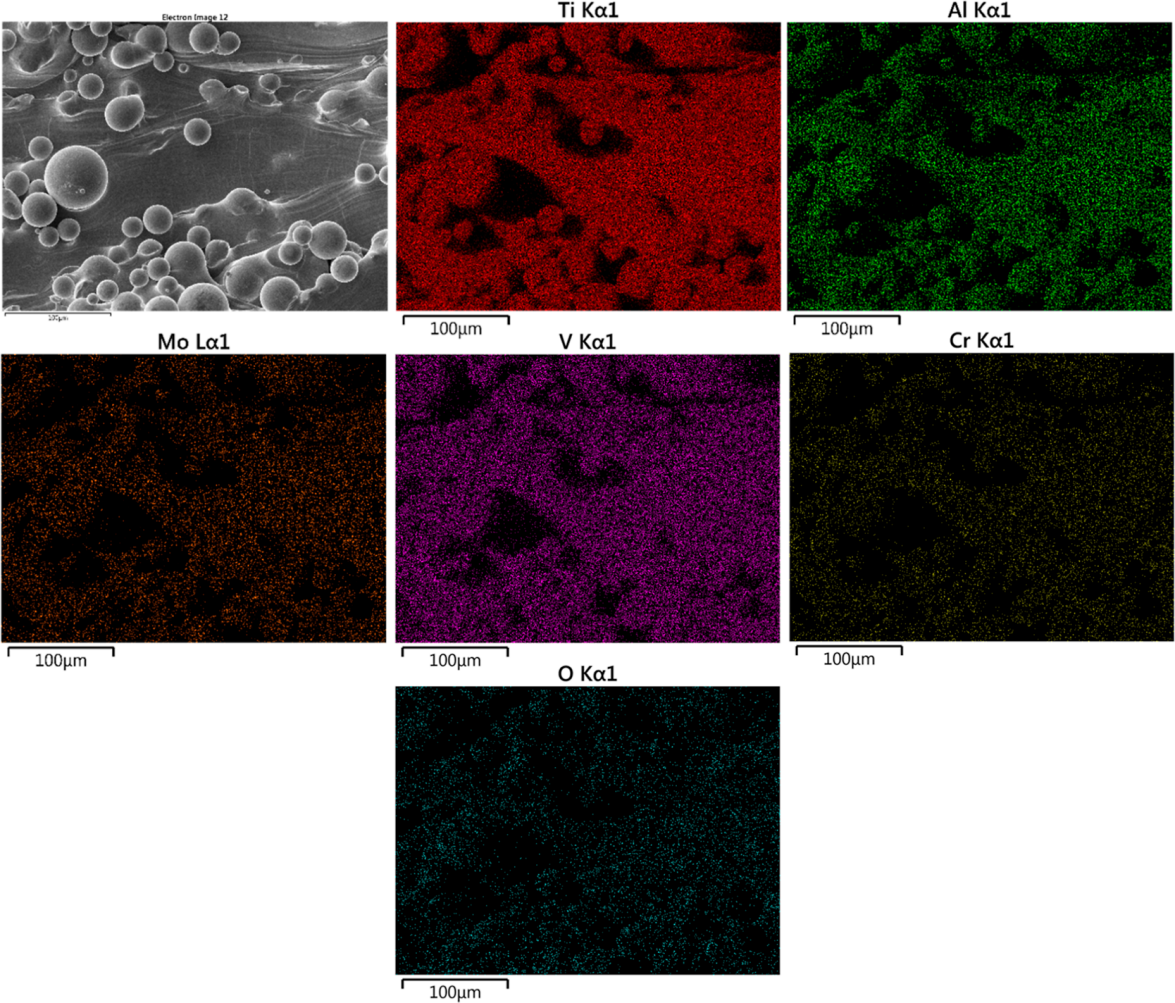
SEM micrograph (secondary electron image) of an as-processed specimen. The *XZ* plane is shown. The EDXS elemental maps for Ti, Al, Mo, V, Cr, and O were obtained from the same area shown in the micrograph.

Figure 5 presents an SEM micrograph for an as-processed Ti-5553 specimen along with associated energy-dispersive X-ray spectroscopy (EDXS) elemental maps for Ti, Al, Mo, V, Cr, and O from the imaged area. Semiquantitative X-ray analysis revealed weight percents of the alloying elements as the following: Al (4.1%), Mo (4.4%), V (5.3%), and Cr (2.1%) were close to the expected weight percents of Al (5%), Mo (5%), V (5%), and Cr (3%). The measured Ti content was 78%, while the measured O content (5.6%) arises from the surface oxide layer. The maps agree with the expected alloy composition.

**Figure 6:**
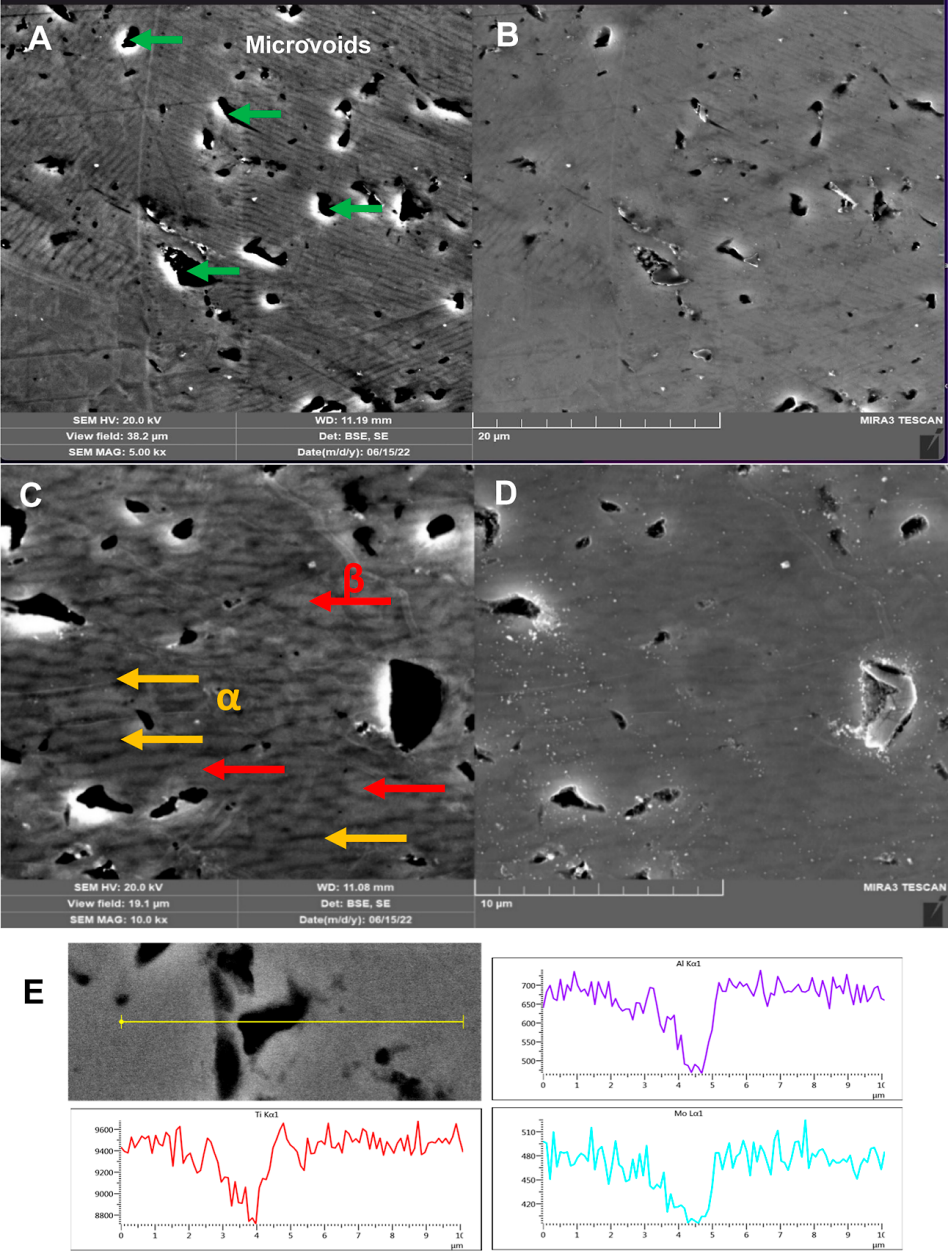
SEM micrographs of the same area of a Ti-5553 specimen (*XZ* plane) in the (A) backscattered electron and (B) secondary electron imaging modes at 5000× (scale bar = 20 μm). SEM micrographs of the same area in the (C) backscattered electron and (D) secondary electron image modes at 10,000× (scale bar = 10 μm). The specimen was wet-abraded on P1500 grit aluminum oxide grinding paper for 4 min followed by ultrasonic cleaning in ultrapure water for 20 min; polished with a 0.25 μm alumina/H_2_O slurry on a polishing wheel for 30 min; rinsed with ultrapure water; polished with a 0.04 μm colloidal silica/H_2_O slurry on a polishing wheel for 40 min; rinsed with ethanol rubbed on a felt pad wetted with ethanol to remove polishing debris; and ultrasonically cleaned in ethanol for 10 min. The specimen was then etched in Kroll’s solution (see Section 2.7). The β phase is brighter, while the α phase is darker in the BSE images. (E) EDXS line spectra collected across the surface that included a microvoid feature.

The SEM micrographs (backscattered electron images) of a Ti-5553 specimen after abrading, polishing, and wet chemical etching (see Section 2.7) are presented in Figure 6. These micrographs inform more about the alloy microstructure. Figure 6A,B reveals some irregularly shaped microvoids and circular pores across the surface (*XZ* plane). The voids are native to the alloy, while some of the pores are likely localized corrosion pits formed during the etching step. Areas consisting of heavier elements (higher atomic number) appear brighter in BSE SEM micrographs, while areas with a higher amount of lighter elements (lower atomic number) appear darker.

Higher-magnification SEM micrographs (secondary and backscattered electron images) for an abraded, polished, and etched specimen (XZ plane) are presented in Figure 6C,D. Pores and microvoids are evident. Some of these are native to the material, and some were likely introduced during the wet chemical etching with Kroll’s solution. Dark rodlike regions, marked by the yellow arrows, are believed to represent the α crystallographic phase (close-packed hexagonal). The brighter regions, marked with red arrows, are believed to be the β phase [body-centered cubic (BCC)]. The fine α precipitates that are dispersed within the β matrix serve to strengthen the alloy. The EDXS line scan analysis data presented in Figure 6E shows the colocalization of Al and Mo within the Ti across the abraded, polished, and wet-etched alloy surface.

### 3.3 X-ray Diffraction Analysis

**Figure 7:**
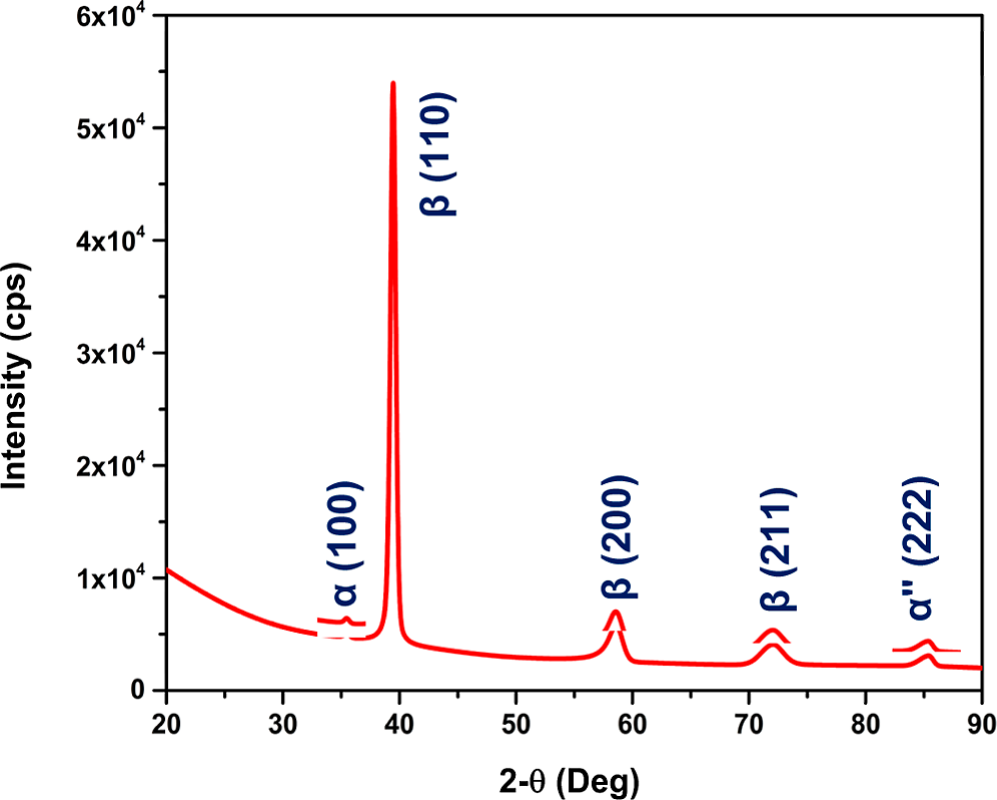
X-ray diffraction pattern of an as-processed Ti-5553 alloy specimen.

An X-ray diffraction spectrum for a typical as-processed Ti-5553 specimen is shown in Figure 7. High-intensity and symmetric diffraction peaks corresponding to the β phase were present at 2θ values (deg) of 39.45 (172,771 counts, 0.52 deg fwhm), 58.54 (10,855 counts, 1.47 deg), and 72.02 (6248 counts, 2.19 deg fwhm).^61,62^ The weakly intense, symmetric peak at a 2θ value of 35.45 deg (1632 counts, 0.49 deg fwhm) and asymmetric peak at a 2θ value of 85.40 deg (3254 counts, 1.33 deg fwhm) are associated with α and α″ phases, respectively. The peak assignments are based on comparison of diffraction pattern data for this alloy.^2,6,47,52,63^ The high intensity of the peak at a 2θ of 39.45 deg indicated that the alloy has a considerable amount of β phase. This is expected because this alloy consists of relatively high atomic levels of the β phase stabilizing elements Mo, V, and Cr.

### 3.4 Vickers Microhardness

The microhardness data revealed good reproducibility across multiple points in a single specimen and from specimen to specimen (*n* = 5). The nominal hardness over three different 2.54 cm × 2.54 cm specimens was 292 ± 2 HV (dwell time = 15 s. Load = 200 gf). This hardness is lower than the value reported in the literature for heat-treated, die-cast Ti-5553 specimens (i.e., 311 ± 8 HV).^14,15,63,64^ The slightly lower value for the SLM specimens is attributed to the estimated 1% porosity. Microvoids and pores observed for these SLM Ti-5553 alloys are not typically present in the fully dense die-cast material. Increasing hardness for SLM Ti-5533 alloys has been correlated with increasing aging temperature.^14,15,63,64^

### 3.5 Electrochemical Properties of As-Processed and Abraded and Polished Alloys

**Figure 8:**
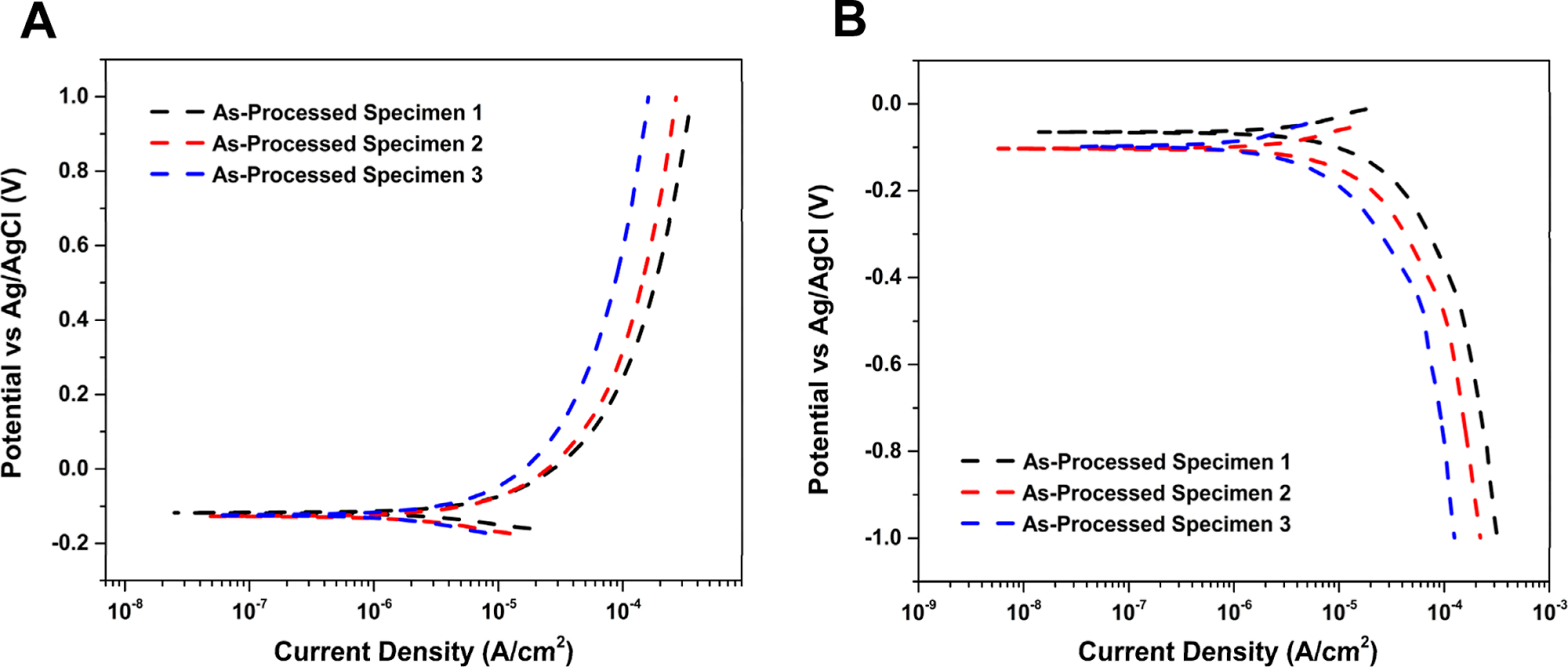
(A) Anodic and (B) cathodic potentiodynamic polarization curves for as-processed Ti-5553 specimens (*XZ* plane) in naturally aerated 3.5 wt % NaCl. Scan rate = 1 mV/s. Three specimens were used for both the anodic and cathodic measurements. The specimens were only degreased and deoxidized prior to the measurements. The currents are normalized to the geometric area of the specimens exposed to the electrolyte solution.

Potentiodynamic polarization curves, both anodic and cathodic, were recorded for multiple as-processed Ti-5553 alloys to assess the reproducibility of the electrochemical properties from specimen to specimen. The specimens were pretreated by only degreasing and deoxidation, as indicated in Section 2.8. Figure 8 presents (A) anodic and (B) cathodic polarization curves for three separate specimens. The same specimens were used to record both curves with the cathodic curves being recorded first. As can be seen, the curve shapes, and therefore the electrochemical properties, were reproducible from specimen to specimen. The anodic curves (Figure 8A) were scanned from 0.050 V negative of the OCP out to 1.0 V vs Ag/AgCl. The current increases significantly with increasing potential immediately positive of the OCP before reaching a relatively constant value. A steady-state current is approached by ca. 0.4 V for all three specimens with a value of ca. 1 × 10^–4^ A/cm^2^ at 0.8 V. Recall that the current is normalized to the geometric and not the true surface area, so the actual current density in this region is less. The steady-state current is associated with the formation of a passivating oxide film (TiO_2_). Below 0.2 V, the alloy was actively oxidizing to form Ti^4+^ ions that then react with H_2_O in the interfacial layer to form TiO_2_ (Ti + 2H_2_O → TiO_2_ + 4H^+^ + 4e^–^).^65^ The current in this aggressive electrolyte is stable to at least 1.0 V with no evidence of oxide film breakdown or initiation of stable pit formation and growth. In fact, some specimens were polarized out to 1.8 V with no oxide film breakdown observed (data not presented). In other words, well-defined transition from passive to active behavior was not observed up to 1.0 V vs Ag/AgCl in this high chloride electrolyte.

The cathodic curves (Figure 8B) were recorded from 0.050 V positive of the OCP down to −1.0 V vs Ag/AgCl. The cathodic current increases with increasing negative potential before reaching a near steady-state current by −0.6 V. The current at this potential is associated with the diffusion-controlled reduction of dissolved oxygen. This was confirmed by observing a decrease in the steady-state current at these potentials after the electrolyte solution was deaerated with N_2_ gas. Some of the current at more negative potentials in this neutral pH electrolyte is likely due to the reduction of the passivating TiO_2_ to hydrated Ti(OH)_3_ [TiO_2_ + 2H_2_ O^+^ e^–^ → Ti(OH)_3_ + OH^–^]. The oxygen reduction reaction on TiO_2_ is complicated and can involve both a 2-electron/2-proton or a 4-electron/4-proton pathway.^66^ Although more research is needed to better understand the oxygen reduction reaction mechanism on this alloy, we suppose the reaction proceeds following the 2-electron/2-proton pathway (1/2O_2_ + H_2_O + 2e^–^ → 2OH^–^).

**Figure 9:**
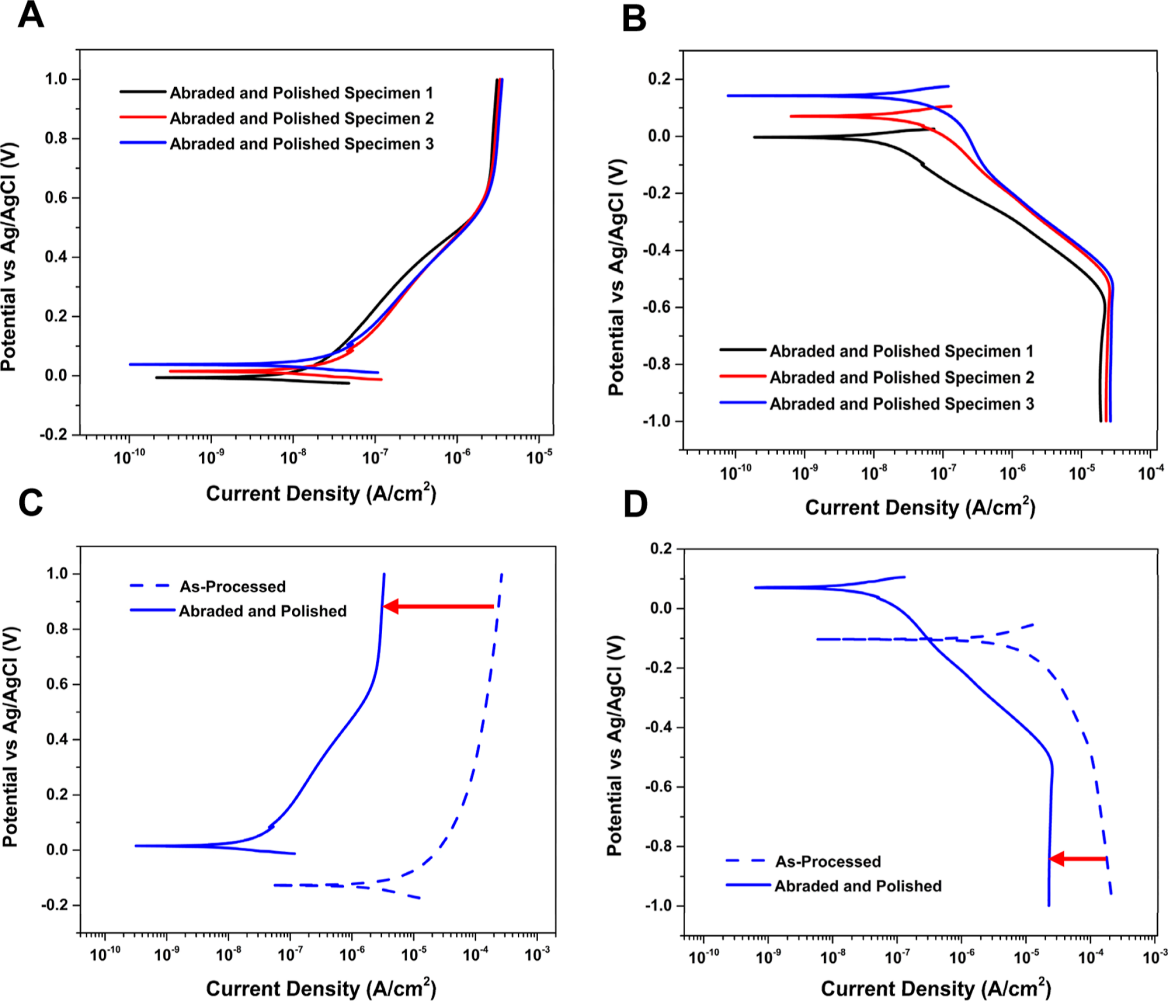
(A) Anodic and (B) cathodic potentiodynamic polarization curves for surface-pretreated Ti-5553 specimens (*XZ* plane) in naturally aerated 3.5 wt % NaCl. Scan rate = 1 mV/s. Three specimens were used for both the anodic and cathodic measurements. Overlays of (C) anodic and (D) cathodic potentiodynamic polarization curves are presented for as-processed and surface-pretreated specimens in naturally aerated 3.5 wt % NaCl. Scan rate = 1 mV/s.

The impact of smoothing the rough alloy surface on the electrochemical properties was investigated. Figure 9A,B presents anodic and cathodic curves for three separate abraded and polished Ti-5553 specimens. The curve shapes and current magnitudes from specimen to specimen were again reproducible. In the anodic potentiodynamic polarization curves (Figure 9A), as the potential is scanned positive, there is a more gradual increase in the anodic current up to about 0.6 V than was observed for the as-processed specimens. As can be seen, a steady state is reached at 0.6 V with a value of ca. 2 × 10^–6^ A/cm^2^. This current density is two orders of magnitude lower than that for the as-processed specimens at the same potential, in part because of the reduced roughness factor (true area/geometric area). No breakdown of the passivating oxide film and onset of localized pit formation and growth was observed on any of three specimens out to at least 1.0 V vs Ag/AgCl. The smoothed specimens are passivated by a low defect, electrochemically formed oxide layer (TiO_2_) that is likely several nanometers thick. The oxide formed on the smoothed surface better passivates the alloy than does the oxide layer formed on the rough as-processed surface, making the surface-pretreated alloy more resistant to corrosion. This is because of a reduced surface area and the fact that the passivating oxide layer can form with fewer defects on a smoother surface. The alloying elements are dissolved in the Ti matrix. However, over time, the dissolution of the alloying elements may impact the corrosion resistance by degrading the overall integrity of the oxide.^66^ As the potential was scanned toward lower values in the cathodic polarization curves (Figure 9B), there is a gradual increase in current up to about −0.5 V, at which point a steady-state current is reached. The reproducible current magnitude at this potential is ca. 2 × 10^–5^ A/cm^2^. Again, this current is associated with dissolved oxygen reduction.

Figure 9C overlays anodic potentiodynamic polarization curves for representative as-processed and surface-pretreated specimens in naturally aerated 3.5 wt % NaCl. A steady-state passivation current is reached at 0.6 V for both specimens, but the current magnitude for the as-processed specimen, ca. 1 × 10^–4^ A/cm^2^, is 100× larger than the magnitude for the surface-pretreated specimen, ca. 2 × 10^–6^ A/cm^2^. Additionally, the OCP value for the as-processed specimen is about 100 mV less noble (more negative) than the value for the surface-pretreated specimen. These data are consistent with the surface-pretreated (abraded and polished) specimens being better passivated by the electrochemically formed oxide layer than the rougher as-processed specimens.

Figure 9D overlays cathodic potentiodynamic polarization curves for replicate as-processed and surface-pretreated specimens in naturally aerated 3.5 wt % NaCl. This current for the reduction of dissolved oxygen on the as-processed specimen is about 2 × 10^–4^ A/cm^2^. In contrast, the current for the surface-pretreated specimen is an order of magnitude lower at ca. 2 × 10^–5^ A/cm^2^. The higher surface area of the as-processed specimen leads to a higher rate of oxygen reduction, as there are more active sites available for the reaction to occur.

Numerical electrochemical data obtained from the polarization curves are summarized in Table 3. The nominal OCP value for the surface-pretreated specimens is more noble by 200 mV than the value for the as-processed specimens. The nominal polarization resistance (*R*_p_) value is larger by 426× for the surface-pretreated specimens at 1.99 (±0.77) × 10^6^ Ω-cm^2^ versus 4.67 (±1.78) × 10^3^ Ω-cm^2^ for the as-processed specimens. The nominal anodic current in the polarization curves at 0.8 V for the surface-pretreated specimens is 72× lower than the value for the as-processed specimens, 2.92 (±0.19) × 10^–6^ versus 2.10 (±0.77) × 10^–4^ A/cm^2^, respectively. Finally, the nominal cathodic current in the polarization curves at −0.8 V for the surface-pretreated specimens is 8× lower than the value for the as-processed specimens, 2.26 (±0.38) × 10^–5^ vs 1.75 (±0.77) × 10^–4^ A/cm^2^, respectively. Therefore, smoothing the surface has a bigger impact on the anodic than on the cathodic current.

**Table 3:** Summary of Electrochemical Parameters for As-Processed and Surface-Pretreated Ti-5553 Specimens in Naturally Aerated 3.5 wt % NaCl^a^

	as-processed	surface-pretreated
OCP (mV vs Ag/AgCl)	–118 ± 7	107 ± 26
*R*_p_—LPR (Ω-cm^2^)	4.67 (± 1.78) × 10^3^	1.99 (± 0.77) × 10^6^
*j* at 0.8 V (A/cm^2^)	2.10 (± 0.77) × 10^–4^	2.92 (± 0.19) × 10^–6^
*j* at −0.8 V (A/cm^2^)	1.75 (± 0.77) × 10^–4^	2.26 (± 0.38) × 10^–5^

a Data are presented as mean ± std. dev for *n* ≥ 3 alloy specimens. Potentials are reported versus Ag/AgCl (4 M KCl).

**Figure 10:**
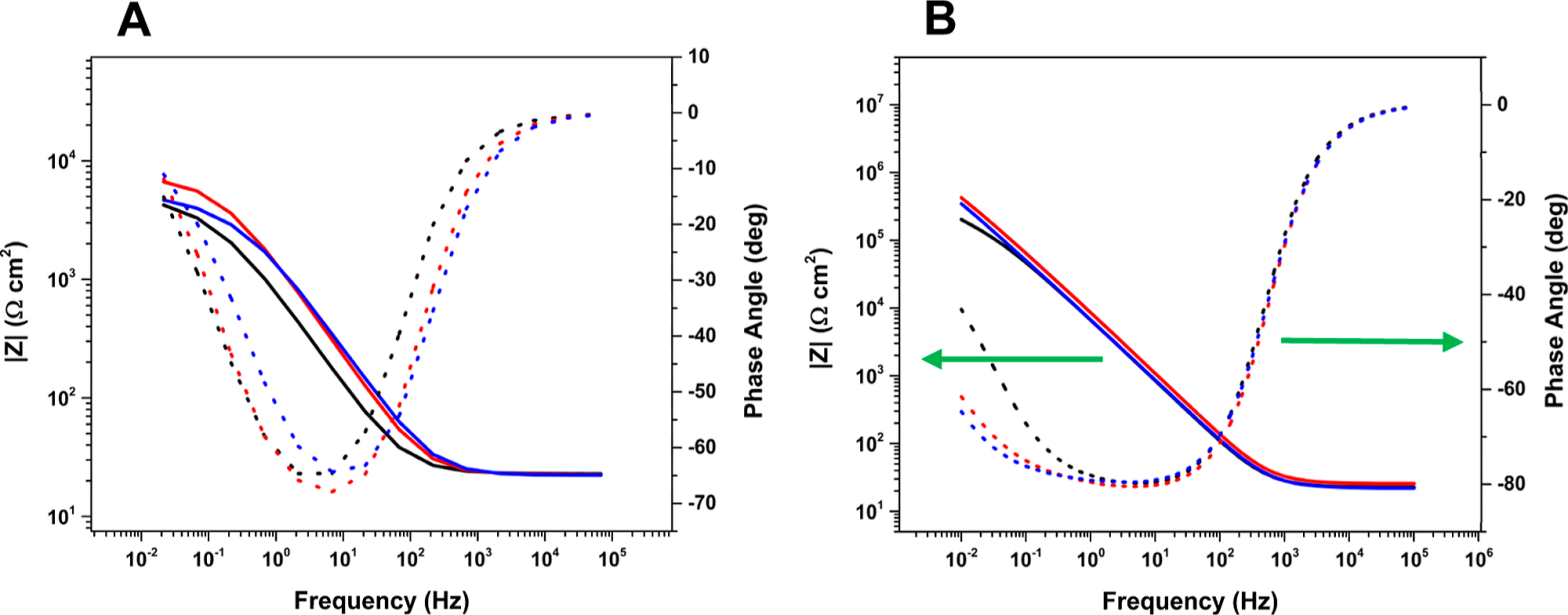
Bode plots of the electrochemical impedance data recorded at the OCP for replicate Ti-5553 specimens: (A) as-processed and (B) surface-pretreated in naturally aerated 3.5 wt % NaCl. All specimens were degreased and deoxidized similarly. AC amplitude = 0.010 V.

EIS measurements were performed at the OCP on the as-processed and surface-pretreated Ti-5553 specimens. Figure 10 presents impedance spectra in the form of Bode diagrams for (A) as-processed and (B) surface-pretreated specimens. A high degree of reproducibility is seen in the replicate curves for each specimen type. At high frequencies, the Bode diagrams for both specimen types exhibit a constant impedance of about 20 Ω cm^2^ with a phase angle near 0°. This corresponds to the series resistance, which is the sum of the ohmic resistances of the metal alloy and electrolyte solution. At middle frequencies, the impedance increases linearly with decreasing frequency while the phase shift approaches −70° for the as-processed and −80° for the surface-pretreated specimens. The slope of the log *Z* vs log frequency plot is close to −1. These trends are reflective of ideal capacitive behavior of the surface oxide film. The larger phase angle for the surface-pretreated specimens and the fact that the phase angle remains near −80° to lower frequencies are consistent with the formation of a more compact and less defective oxide layer on these specimens.

At low frequencies, the phase angle decreases toward 0°, and the impedance reaches a maximum. Importantly, the magnitude of the impedance at 0.01 Hz (see Table 4) is 35× larger for the surface-pretreated specimens as compared to the value for as-processed specimens, 1.84 (±0.49) × 10^5^ vs 5.19 (±1.29) × 10^3^ Ω-cm^2^, respectively. The low-frequency impedance is reflective of the polarization or charge-transfer resistance and, therefore, is a measure of the corrosion resistance of the alloy. The increased low-frequency impedance for the surface-pretreated specimens is consistent with the trends in *R*_p_ and the anodic and cathodic polarization currents, all reflective of increased alloy corrosion resistance after smoothing the surface texture.

**Table 4:** Summary of EIS Parameters Determined from Fitting the Experimental Data for Ti-5553 Alloy Specimens as Processed and after Surface Pretreatment in Naturally Aerated 3.5 wt % NaCl^a^

	as-processed	surface-pretreated
polarization resistance—*R*_p_ (Ω-cm^2^)	5.21 (± 1.33) × 10^3^	1.66 (± 0.97) × 10^6^
equivalent series resistance—*R*_s_ (Ω-cm^2^)	22.20 ± 0.40	24.30 ± 1.98
effective capacitance—*C*_eff_ (F/cm^2^)	5.29 (± 2.61) × 10^–5^	1.05 (± 0.12) × 10^–5^
impedance modulus—*Z*_Mod_ at 0.01 Hz (Ω-cm^2^)	5.19 (± 1.29) × 10^3^	1.84 (± 0.49) × 10^5^

a Data are presented as mean ± std. dev. for *n* = 3 specimens of each type. EIS measurements were made at the OCP. χ^2^ values for the as-processed and the surface-pretreated (abraded and polished) specimens 5.1 × 10^–4^ and 4.2 × 10^–4^, respectively.

The EIS data were fit to a simple Randles equivalent circuit consisting of an equivalent series resistance (*R*_s_) in series with the parallel combination of a constant phase element, in place of a capacitor, and a polarization resistance (*R*_p_). The equivalent series resistance is the sum of the ohmic resistance of the electrolyte solution, electrode, and electrical contact. These values were then used to calculate the effective capacitance using the following equation^67^
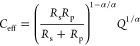
1

The CPE components were expressed by mathematical parameters, *Q* and α, where *Q* is the quasi-capacitance and α is the so-called homogeneity factor (α = 1 for an ideal capacitor).

Table 4 presents a summary of the electrochemical parameters extracted from the EIS data. The nominal *R*_p_ value for the surface-pretreated specimens is 318× larger than the value for the as-processed specimen, 1.66 (±0.97) × 10^6^ vs 5.21 (±1.33) × 10^3^ Ω-cm^2^. These values are consistent with the *R*_p_ values determined from the LPR measurements presented in Table 3 and reflect the increased corrosion resistance of the alloy with a reduced surface roughness. The equivalent series resistance, *R*_s_, is the same for both specimens. This is expected because the magnitude of *R*_s_ is dominated by the electrolyte resistance. The effective capacitance, *C*_eff_, is lower for the surface-pretreated specimens, 1.05 (±0.12) × 10^–5^ vs 5.29 (±2.61) × 10^–5^ F/cm^2^. This is due to the improved dielectric properties of the passivating oxide layer on the surfaces of pretreated specimens. For example, a thicker and more continuous dielectric oxide layer would reduce *C*_eff_.

### 3.6 Effect of Deoxidation on the Electrochemical Properties

Since both specimen types were degreased and deoxidized similarly with commercial solutions, we conducted some preliminary experiments to learn how the electrochemical properties of the alloy are impacted. The deoxidizer or desmutter employed was designed for use with aluminum alloys to dissolve smut and other contaminants from the surface. It is unclear how these wet chemical treatments impact the electrochemical properties of this alloy. Smut contaminants can negatively impact the formation of coatings and surface finishes used to mitigate corrosion and therefore need to be removed. This can be done with a deoxidizing acidic bath, such as the Smut-Go bath used herein.

The alloy specimens were first smoothed by abrading on P1500 grit aluminum oxide grinding paper for 20 min using a polishing wheel followed by ultrasonic cleaning in ultrapure water for 30 min. The specimens were then polished (by hand) using 1 and 0.3 μm alumina powder/H_2_O slurries for 30 min each. After each polishing step, the specimens were ultrasonically cleaned in ultrapure water for 30 min. As a final step, the specimens were polished with a 0.05 μm alumina powder/H_2_O slurry, followed by ultrasonic cleaning in ultrapure water for 30 min. One set of three received no additional surface pretreatment (i.e., no degreasing and deoxidation) prior to the electrochemical measurements. Another set of three was degreased and deoxidized (2 min), as described in the experimental section, prior to the electrochemical measurements. Table 5 presents a summary of electrochemical data obtained from the OCP measurements, anodic and cathodic potentiodynamic polarization curves, and linear polarization resistance measurements in naturally aerated 3.5 wt % NaCl at room temperature. This data reveals that the smut-go deoxidation has a minor effect on the electrochemical behavior of this alloy, at least for the conditions employed. There is a noble shift in the OCP and 2× increase in the nominal *R*_p_. The anodic and cathodic polarization curve currents (naturally aerated) at the selected potentials are 2× lower than the values for the specimens that were degreased and deoxidized, consistent with the increase in *R*_p_. The numerical data presented in Table 5 agree with those presented in Table 3. Future work will examine the electrochemical properties in more detail as well as morphology, microstructure, and surface chemistry of the alloy after different deoxidation times.

**Table 5:** Summary of Electrochemical Parameters for Surface-Pretreated Ti-5553 Specimens before and after Degreasing and Deoxidation in Naturally Aerated 3.5 wt % NaCl^a^

electrode	OCP (mV vs Ag/AgCl 3 M KCl)	*j* at 0.6 V (A/cm^2^)	*j* at −0.6 V (A/cm^2^)	*R*_p_ (Ω cm^2^) × 10^6^
surface-pretreated (naturally aerated)	–214 ± 49	8.97 (±0.48) × 10^–6^	7.85 (±0.94) × 10^–5^	0.85 ± 0.48
surface-pretreated (deaerated)	–458 ± 41	5.49 (±0.86) × 10^–6^	3.61 (±0.45) × 10^–5^	1.30 ± 0.21
surface-pretreated plus degreased and deoxidized (naturally aerated)	–25 ± 6	4.02 (±1.06) × 10^–6^	3.17 (±0.51) × 10^–5^	1.75 ± 0.04

a Data are presented as mean ± std. dev. for *n* = 3 alloy specimens of each type.

## 4 Discussion

Given the absence of literature reports on the electrochemical properties of Ti-5553 alloys prepared by SLM processing, the results reported herein are important and fill a knowledge gap. In this work, the electrochemical properties of alloy specimens as-processed and surface-pretreated to renew and smooth the surface texture were investigated. Metal alloys prepared by SLM tend to possess a significant surface roughness. The surface roughness can vary depending on several factors including the SLM processing parameters, powder characteristics, laser heating parameters, and postprocessing techniques. The as-processed specimens used in this work had surface roughness, *S*_q_, and maximum peak-to-valley height, *S*_z_, values of 16.7 ± 5.3 and 111 ± 29 μm, respectively (*n* = 5 spots on three specimens). The specimens were comprised of balling features with some micropore and fusion pore defects. After abrading and polishing, the *S*_q_ and *S*_z_ values decreased to 1.8 ± 0.1 and 6.4 ± 0.4 μm, respectively. Given the hardness and density of this alloy, 292 ± 2 HV and 4.62 ± 0.04 g/cm^3^, respectively, smoothing the surface texture is most effectively accomplished by using a polishing wheel with a series of decreasing alumina grit sizes. A key finding from the work is that reducing the surface roughness by abrading and polishing improves the corrosion resistance of the alloy. In general, Ti and Ti alloys have excellent resistance against corrosion, even in concentrated Cl^–^ electrolyte solutions. They are susceptible, however, to erosion corrosion, stress corrosion cracking, corrosion fatigue, and crevice corrosion. The corrosion resistance of Ti and its alloys is due to the formation of a protective and electrically insulating oxide layer, consisting of TiO_2_, with a morphology that depends on the surface condition of the alloy on which it forms.^66,68^ The improved corrosion resistance of the alloy after smoothing is attributed to the removal of the native and more defective oxide film and reformation of a more compact and less defective oxide. The surface area of the metal alloy exposed to the solution is also reduced after abrading and polishing. This also serves to make the alloy more corrosion-resistant. Reducing the surface area will produce lower corrosion rates due to decreased reaction site density, decreased mass transport pathways, and diminished susceptibility to localized corrosion processes.

A priori, native defects and surface microstructure are expected to impact the electrochemical properties and corrosion susceptibility of the Ti-5553 alloy. Microvoids and pores are defects inherent to the SLM alloy because of incomplete annealing and particle fusion.^69^ These defects represent sites where the solution could penetrate the alloy and cause localized corrosion. These are also sites where there would be incomplete coverage of the passivating oxide layer. Some microvoids and pores were revealed on the alloy specimens used in this work; although from a bulk perspective, the specimens are nearly fully dense based on comparison of the measured density with the value reported for the die-cast alloy. A ca. 1% porosity is estimated for these SLM alloys, although some of the pores seen in the electron micrographs are likely introduced during the smut-go deoxidation treatment. This needs to be investigated further, as these defects will deleteriously impact the formation of coatings and other surface finishes applied for corrosion mitigation on this alloy.

A detailed microstructural characterization of the SLM Ti-5553 alloys was not part of this work, but a significant body of literature exists on this. The crystal structure of pure Ti at ambient temperature and pressure is close-packed hexagonal, known as the α phase. At about 890 °C, the Ti undergoes an allotropic transformation to a BCC structure, known as the β phase.^8,9,13−15,61−63^ This β phase remains stable at the melting temperature (1700 °C). The alloying elements can be categorized according to their stabilizing effect on the α and β phases.^8,9,13−15,61−63^ Some alloying elements, such Al, are α stabilizers, while other elements, such as Mo, Nb, Ta, V, and Cr, are β stabilizers. The presence of the α and β phases depends on the relative amounts of the respective stabilizers. Ti-5553 is a near-β-phase alloy consisting of some α phase within a matrix of the β phase. Literature shows that Ti-5553 can be multidirectionally forged in the two-phase α + β field that consists of a microstructure with large globular and rodlike, needle-shaped α precipitates within the β phase matrix.^61−63,70^ XRD data for Ti-5553 specimens used in this work indicate a largely β-phase alloy based on the higher intensity of the β-phase peaks relative to the α-phase ones.

The improved corrosion resistance of the surface-pretreated Ti-5553 alloys is supposed to result from the formation of a stable, continuous, adherent, and protective oxide film on the surface. The surface oxide film, typically less than 10 nm, forms spontaneously and instantly when fresh metal surfaces are exposed to air and/or moisture. Of course, the oxide can also be formed electrochemically. The composition and characteristics of the oxide layer that forms on the alpha and beta phases of titanium can vary due to differences in the crystal structure and chemical composition. In alpha-phase titanium (hexagonal close-packed), the oxide layer typically consists of primarily TiO_2_, with variations in the crystal structure, such as rutile and anatase. Beta-phase titanium (BCC) also forms TiO_2_ as the predominant oxide, but the specific morphology and thickness of the oxide layer may differ due to the different crystallographic arrangement of alloying elements present in the beta phase. Overall, TiO_2_ remains the primary oxide formed on both alpha and beta phases of titanium, providing corrosion resistance and other beneficial properties. Alloying elements at the surface will influence the elemental composition of the surface oxide. For example, Al (5 wt %) and V (5 wt %) are expected to form stable Al_2_O_3_ and V_2_O_5_ oxide layers.^71^ Discontinuities in the oxide layer are expected to exist at the interface of the alloy element secondary phases and the surrounding Ti matrix. These represent defect sites through which ions in solution could penetrate the oxide and reach the underlying metal.^72,73^

## 5 Conclusions

The electrochemical properties of the Ti-5553 alloy prepared by SLM fabrication were investigated before and after surface pretreatment by abrading and polishing. Abrading and polishing reduce the surface roughness, producing a surface oxide finish that renders Ti-5553 more resistant to electrochemical corrosion due to the removal of the defective native oxide on the as-processed specimens and the formation of a more compact and less defective oxide film on the refreshed and smoothed surfaces. Additionally, the abrading and polishing reduce the surface area exposed to the solution, leading to a diminished corrosion rate. Preliminary results indicate that the deoxidizing Smut-Go pretreatment, designed for aluminum alloys, of the smoothed alloy has a minimal impact on the electrochemical behavior of this Ti alloy.

The key findings from this work are summarized as follows:

- The surface roughness, *S*_q_, and maximum peak-to-valley height, *S*_z_, values of the as-processed specimens were 16.7 ± 5.3 and 111 ± 29 μm, respectively (*n* = 5 spots on three specimens). The specimens were comprised of balling features as well as some micropore and fusion pore defects. After abrading and polishing, *S*_q_ and *S*_z_ decreased to 1.8 ± 0.1 and 6.4 ± 0.4 μm, respectively.
- The nominal density of the as-processed specimens was 4.62 ± 0.04. Based on comparison with the reported density for the die-cast alloy, the specimens were 99–100% dense with an estimated 1% porosity. Vickers microhardness measurements revealed a nominal value of 292 ± 2 HV, which is slightly lower than the reported value for the die-cast alloy of 311 ± 8 HV.
- The OCP for the surface-pretreated specimens in naturally aerated 3.5% NaCl was more noble (or positive of the value) for the as-processed specimens, 107 ± 26 vs −118 ± 7 mV vs Ag/AgCl (4 M NaCl). The nominal polarization resistance (*R*_p_) obtained from linear polarization resistance measurements for the surface-pretreated specimens was 426× higher than the value for the as-processed specimens, 1.99 (±0.77) × 10^6^ vs 4.67 (±1.78) × 10^3^ Ω-cm^2^. Both are consistent with the corrosion resistance of the alloy being improved by the abrading and polishing.
- Anodic potentiodynamic polarization curves revealed a nominal anodic current of 2.10 (±0.77) × 10^–4^ A/cm^2^ at 0.8 V vs Ag/AgCl (4 M NaCl) for the as-processed specimens. This value decreased by 72× to 2.92 (±0.19) × 10^–6^ A/cm^2^ for the surface-pretreated specimens. The nominal cathodic current in the potentiodynamic polarization curves at −0.8 V was 1.76 (±0.77) × 10^–4^ A/cm^2^ and decreased by 8× after surface pretreatment to 2.26 (±0.38) × 10^–5^ A/cm^2^. Both indicate reduced oxidation and reduction reaction rates.
- EIS data revealed a nominal low-frequency impedance modulus at 0.01 Hz that was 35× larger and a polarization resistance from equivalent circuit fitting that was 318× larger (1.7 × 10^6^ vs 5.2 × 10^3^ Ω-cm^2^) for the surface-pretreated specimens compared to the rougher as-processed specimens. This is reflective of the improved corrosion resistance after smoothing the alloy surface.
